# Molecular and clinical significance of fibroblast growth factor 2 (FGF2 /bFGF) in malignancies of solid and hematological cancers for personalized therapies

**DOI:** 10.18632/oncotarget.8203

**Published:** 2016-03-19

**Authors:** Mohamed R. Akl, Poonam Nagpal, Nehad M. Ayoub, Betty Tai, Sathyen A. Prabhu, Catherine M. Capac, Matthew Gliksman, Andre Goy, K. Stephen Suh

**Affiliations:** ^1^ Genomics and Biomarkers Program, The John Theurer Cancer Center, Hackensack University Medical Center, Hackensack, NJ, USA; ^2^ Department of Clinical Pharmacy, Faculty of Pharmacy, Jordan University of Science and Technology, Irbid, Jordan; ^3^ Lymphoma Division, The John Theurer Cancer Center, Hackensack University Medical Center, Hackensack, NJ, USA

**Keywords:** bFGF, FGF2, diagnosis, prognosis, malignancy

## Abstract

Fibroblast growth factor (FGF) signaling is essential for normal and cancer biology. Mammalian FGF family members participate in multiple signaling pathways by binding to heparan sulfate and FGF receptors (FGFR) with varying affinities. FGF2 is the prototype member of the FGF family and interacts with its receptor to mediate receptor dimerization, phosphorylation, and activation of signaling pathways, such as Ras-MAPK and PI3K pathways. Excessive mitogenic signaling through the FGF/FGFR axis may induce carcinogenic effects by promoting cancer progression and increasing the angiogenic potential, which can lead to metastatic tumor phenotypes. Dysregulated FGF/FGFR signaling is associated with aggressive cancer phenotypes, enhanced chemotherapy resistance and poor clinical outcomes. *In vitro* experimental settings have indicated that extracellular FGF2 affects proliferation, drug sensitivity, and apoptosis of cancer cells. Therapeutically targeting FGF2 and FGFR has been extensively assessed in multiple preclinical studies and numerous drugs and treatment options have been tested in clinical trials. Diagnostic assays are used to quantify FGF2, FGFRs, and downstream signaling molecules to better select a target patient population for higher efficacy of cancer therapies. This review focuses on the prognostic significance of FGF2 in cancer with emphasis on therapeutic intervention strategies for solid and hematological malignancies.

## INTRODUCTION

The fibroblast growth factor (FGF) family consists of 23 FGF signaling polypeptides that function as potent mitogens [1–3]. FGFs exert broad mitogenic activity by stimulating the growth of fibroblasts, endothelial, and cancer cells. The family plays important roles in embryonic development, maintenance of adult organ systems, tissue regeneration, wound repair, and hematopoiesis. FGF2, also known as basic FGF (bFGF), is the prototypical and most studied member of the FGF superfamily. FGF2 is an important regulator of cell growth and differentiation under physiological and pathological conditions [1–3]. Previous studies have suggested a role of FGF2 as a prognostic marker for different types of malignancies. This review summarizes the biology of FGF signaling by demonstrating biological roles of FGF2 in regards to pathogenesis and prognosis of solid and hematological tumors with a special focus on clinical development of FGF2 inhibitors in the era of personalized medicine.

### FGF2 isoforms, receptors, binding partners, and signaling

**Figure 1:**
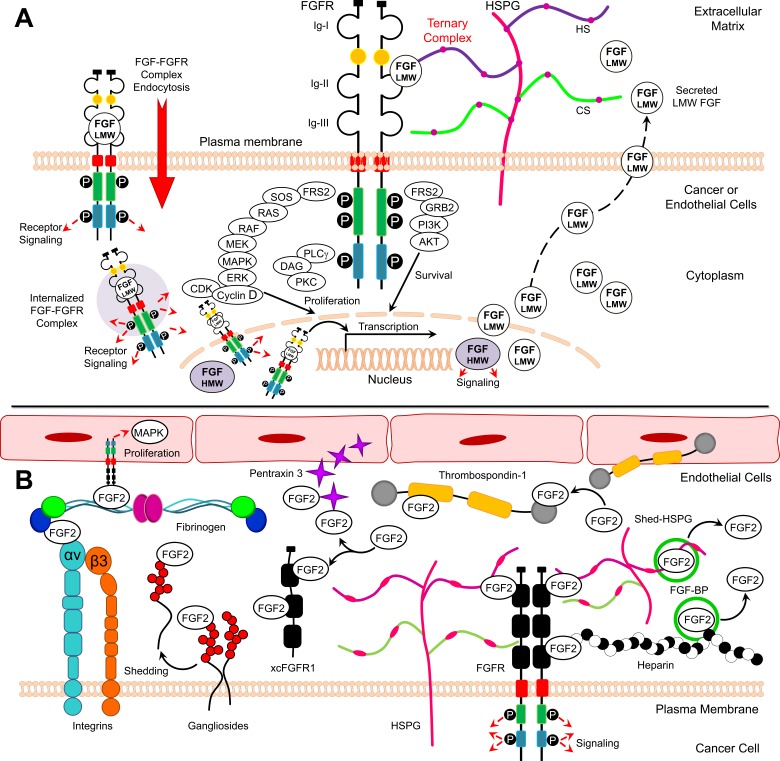
Model for FGF2/FGFR signaling and FGF2 binding partners **A.** Structure of activated FGFR and downstream signaling **B.** FGF2 soluble and membranous binding partners.

FGF2 exists in low and high molecular weight isoforms, which are translated from a single common mRNA through alternative translation-initiation codons [4]. Low molecular weight (LMW) FGF2 is an 18 kDa protein translated from a conventional AUG start codon [5]. LMW FGF2 is found in the cytoplasm and nucleus and can be also secreted by target cells [6]. In order to initiate signaling, LMW FGF2 interacts with cell surface heparan sulfate proteoglycans (HSPGs) and fibroblast growth factor receptor (FGFR) in a ternary complex consisting of FGF2, FGFR, and HSPG resulting in activation of downstream signaling pathways, including Ras, Raf, MAPK and ERK (Figure 1A) [7]. High molecular weight (HMW) FGF2 isoforms (22-, 22.5-, 24-, and 34-kDa) are produced through translation initiation at CUG sites upstream and in frame of the AUG codon. HMW FGF2 localizes to the nucleus and signals independently of FGFRs [8]. Similar to HMW FGF2, LMW FGF2 can also function in the cytosol and nucleus of cells through endocytosis of activated FGF-FGFR complexes [9]. FGFR1 and FGF2 have been shown to co-localize in the nuclear matrix, where together they may co-activate transcription and thus control cell proliferation (Figure 1A) [10, 11].

Five FGFRs have been identified, four of which (1-4) are highly conserved single-pass transmembrane tyrosine kinase receptors [12]. The extracellular regions of these receptors contain three immunoglobulin (Ig)-like domains (designated as IgI, IgII, and IgIII) linked to the cytoplasmic domain *via* a transmembrane α-helix (Figure 1A). FGFRs 1-3 can undergo alternative splicing during gene expression, and the IgIII domain is composed of an invariant IgIIIa exon alternatively spliced to IgIIIb or IgIIIc. The expression of IgIIIb and IgIIIc is important in defining FGF signaling specificity. While FGF1 binds to all FGFRs, FGF2 binds to FGFR1 (IIIb), FGFR1 (IIIc), FGFR2 (IIIc), and FGFR4 [2]. It has been reported that LMW FGF2 predominantly binds to FGFR1 (IIIc) and weakly to the other FGFRs [5, 13]. The cytoplasmic domain of FGFRs 1-4 contains a juxtamembrane split kinase domain, which contains tyrosine kinase motifs and a C-terminal tail [12]. Although FGFR5 lacks intracellular tyrosine kinase domain, this receptor can bind to multiple FGF ligands acting as a negative regulator of signaling [14].

FGF2 utilizes HSPGs, such as syndecans (SDC), as binding partners to stabilize the FGF-FGFR interaction and enhance resistance to proteolysis [15, 16]. FGF2 cannot activate FGFRs in cells lacking heparan sulfate [17]. After the binding of FGF and HSPG to FGFR to form a ternary FGF:FGFR:HSPG complex, FGFRs dimerize leading to conformational changes in FGFR structure and subsequent intermolecular transphosphorylation of multiple cytoplasmic tyrosine residues (Figure 1A) [12, 18]. FGFR transmits extracellular signals to two main intracellular substrates, which are phospholipase C-γ1 (PLC-γ1) (also known as FRS1) and FGFR substrate 2 (also known as FRS2) (Figure 1A). The phosphorylation of FGFR1 tyrosine residues creates binding sites for SH2 domain of PLC-γ required for phosphorylation and activation of PLC-γ. Conversely, FRS2 constitutively associates with the juxtamembrane region of the FGFR. The phosphorylation of FRS2 is essential for activation of the Ras-mitogen-activated protein kinase (MAPK) and phosphoinositide 3-kinase-Akt (PI3K-Akt) signaling pathways in cancer and endothelial cells (Figure 1A) [12, 19].

FGF2 also interacts with immobilized molecules bound to extracellular matrix (ECM), including cell membrane receptors and soluble molecules (Table 1, Figure 1B). The complex interactions between FGF2 and these molecules control bioavailability, stability, and concentration of FGF2 in the microenvironment [20]. FGF2 can tightly bind HSPG in ECM and is only released through the action of FGF-binding protein (FGF-BP), which is a critical controller of FGF bioavailability (Table 1, Figure 1B). In addition, the binding of FGF to heparin, released HSPG, or cell surface-bound HSPG also regulate FGF bioavailability and the interactions with FGFRs (Table 1, Figure 1B). Conversely, thrombospondin-1 (TSP-1) and pentraxin 3 (PTX3) prevent the interaction of FGF2 with cell surface HSPGs and FGFRs. Similarly; xcFGFR1 (a soluble form of the extracellular portion of FGFR1) binds FGF2 and prevents FGF2/FGFR interaction (Table 1, Figure 1B).

**Table 1:** FGF2 binding partners and associated proteins

Associated Protein	Association	Level of Interaction	Cell Type	Pathway	Ref
FGFR(s)	FGF2 binds to extracellular domain of FGFRs which causes receptor dimerization and autophosphorylation of tyrosine kinase residues on cytoplasmic domain	Cell membrane	Endothelial cells, cancer cells, fibroblasts	FGFR	[12, 101]
HSPG	FGF2 binds with low affinity to heparan sulfate chains of HSPG. This interaction can activate intracellular signaling, promote FGF2 internalization, or by presenting FGF2 to FGFR in proper conformation. HSPG also act as reservoirs for FGF2 which protect them from degradation	Cell membrane	Cancer, endothelial cells	FGFR	[102–104]
αvβ3 integrin	FGF2 binds to αvβ3 integrin leads to assembly of focal adhesion plaques	Cell membrane	Endothelial cells	FAK	[105]
Gangliosides	Gangliosides bind FGF2 via Neu-Ac residues and acts as coreceptors	Cell membrane	Endothelial cells	FGFR	[106]
Free gangliosides	Exogenous gangliosides affect the angiogenic activity of FGF2. Exogenous gangliosides act as FGF2 antagonists when added to endothelial cell cultures	Soluble or associated with ECM	Cancer, endothelial cells	FGFR	[107]
Heparin	Short heparin chains bind FGF2, thus interfering with mitogenic signaling through activation of FGFR, relatively longer chains are expected to induce the adverse effect of potentiating the mitogenic signaling	Soluble or associated with ECM	Tumor Cells	FGFR	[108]
xcFGFR1	A soluble form of extracellular domain of FGFR1 which binds to FGF2 which leads to suppression of FGF2/FGFR1 interaction	Soluble	Endothelial ECM	FGFR	[107]
TSP	TSP-1 and TSP-2 regulate angiogenesis through binding and sequestration of FGF2	Soluble or associated with ECM	Cancer, endothelial cells	FGFR	[109, 110]
PTX3	PTX3 prevents FGF2 binding to FGFRs on endothelial cells, leading to inhibition cell proliferation and motility. PTX3 suppressed neovascularization triggered by FGF2 in the chick embryo chorioallantoic membrane	Soluble or associated with ECM	Endothelial cells	FGFR	[111]
Fibrinogen/ fibrin	Binding of FGF2 to fibrinogen or fibrin provides protection of FGF2 from proteolytic degradation. Fibrinogen potentiates FGF2-stimulated proliferation of endothelial cells. Fibrinogen promotes growth of cancer cells through interaction with FGF2	Soluble or associated with ECM	Endothelial cells, cancer cells	FGFR	[112, 113]
α2M	α2M induces FGF2 expression in embryonic stem cells	Nucleus	Embryonic stem cells	ERK1/2, PI3K,	[114]
PDGF	FGF2 stimulates PDGFR-α and β expression in endothelial cells	Nucleus	Endothelial cells	Transcriptional	[115]
PF4	PF4 inhibits FGF2-induced proliferation of endothelial cells. PF-4 binds to FGF2 and inhibits FGF2 dimerization, binding to FGFRs, and internalization	Soluble or associated with ECM	Endothelial cells	ERK	[116, 117]
uPA	FGF2 upregulates uPA receptor and stimulates uPA production by endothelial cells	Nucleus	Endothelial cells	Transcriptional	[118]
CXCL13	CXCL13 chemokine displaces FGF2 binding to endothelial cells, inhibits FGF2 homodimerization, and induces the formation of CXCL13-FGF2 heterodimers	Soluble or associated with ECM	Endothelial cells	FGFR	[119]
IL-6	FGF2 induces IL-6 release in human pancreatic periacinar myofibroblasts. Overexpression of FGF2 (24-kDa isoform) upregulates IL-6 transcription in NIH-3T3 cells. FGF2 is downstream effector of IL-6-induced angiogenic activity in cancer cells	Soluble or associated with ECM/Nucleus	Fibroblasts, cancer cells	IL-6, ERK1/2 and p38 MAP kinases	[120–122]
E-cadherin	FGF2 downregulates E-cadherin expression through the activation of PI3K/Akt/mTOR and MAPK signaling, and upregulates Slug and ZEB1 in human ovarian cancer cells	Nucleus	Cancer, endothelial cells	PI3K/Akt and ERK	[123]
Bcl-2	FGF2 downregulates Bcl-2 and promotes apoptosis in human breast cancer cells	Nucleus	Cancer cells	Transcriptional	[124]
Aquaporin3	Aquaporin3 is required for FGF2-induced cell migration in human breast cancer cells	Soluble or associated with ECM	Cancer cells	Trans-epithelial fluid transport	[125]
Translokin	Translokin interacts specifically with LMW FGF2. Inhibiting Translokin expression by RNA interference reduces the translocation of FGF2	Soluble or associated with ECM	Fibroblasts	FGF2 trafficking	[126]
Thrombin	Thrombin cleaves HMW FGF2 into a LMW FGF2-like form that stimulates endothelial cell migration and proliferation	Soluble or associated with ECM	Endothelial, cancer cells	Endothelial cell migration	[127]
FGF-binding protein	FGF-BP 1 binds FGF2 and enhances FGF2-dependent proliferation of NIH-3T3 fibroblasts and FGF2-induced extracellular signal-regulated kinase 2 phosphorylation	Soluble or associated with ECM	Squamous, epithelial cells, fibroblasts	FGFR	[128]

α2M: α2-macroglobulin; ECM: extracellular matrix; FAK: focal adhesion kinase; HSPG: heparan sulfate proteoglycan; IL-6: interleukin-6; PDGF: platelet-derived growth factor; PF4: platelet factor 4; PTX3: p; TSP: thrombospondin-2; uPA: urokinase-type plasminogen activator

Gangliosides are glycosphingolipids bound to cell membranes regulating growth of a wide variety of normal cells by binding to FGFs. Gangliosides are highly synthesized in metastatic tumors and are known to shed into the ECM. Integrins are transmembrane receptors that regulate the response to soluble FGF2 in endothelial cells. The interaction between αvβ3 integrin and FGF2 promotes endothelial cell proliferation by activating the MAPK pathway [21]. In addition, the binding of fibrinogen to FGF2 requires αvβ3 integrin to promote endothelial cell proliferation (Table 1, Figure 1B). These findings indicate that FGF2 bioactivity and interaction with FGFR is highly regulated by a complex network of interactions with various FGF2 binding partners.

## ROLES OF FGF2 IN TUMOR PROGRESSION

**Figure 2:**
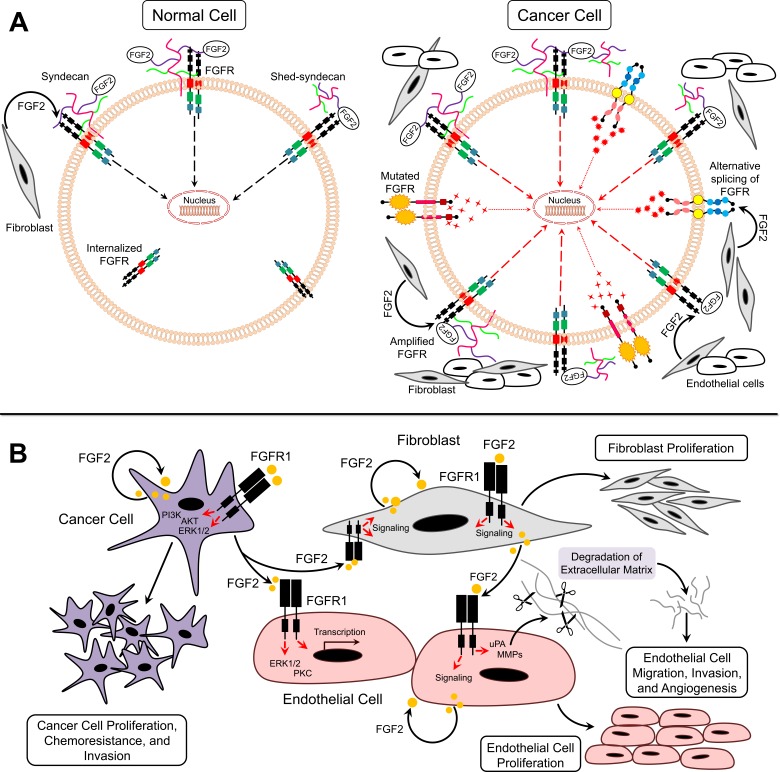
FGF2/FGFR signaling in cancer **A.** Model for FGF2/FGFR function under normal and cancer conditions **B.** Paracrine and autocrine signaling of FGF2 in tumor microenvironment.

Accumulating evidence suggests that FGF2/FGFR signaling is involved in several biological functions, such as embryonic development, tissue regeneration, wound repair, and normal hematopoiesis [1–3]. Expression of FGF2 and FGFRs in normal cells is highly regulated, and termination of FGF2 signaling is achieved through receptor internalization (Figure 2A) [1–3]. However, FGF2/FGFR signaling in cancer cells is dysregulated, which may contribute to the pathogenesis of many types of cancer (Figure 2A). Several studies have shown that FGF2 is a key tumor-promoting factor in the tumor microenvironment. The following section reviews current knowledge of the molecular pathways associated with FGF2 signaling in cancer, which represents a critical step for the implementation of strategies toward the development of personalized cancer therapy.

### Deregulation of FGFR signaling

FGFR amplification and/or upregulation occur in cancer due to deregulated transcription or chromosomal amplification (Figure 2A) [22]. The upregulation of FGFR1 induces cellular transformation of non-transformed cells [23]. FGFR2 upregulation is associated with poor prognosis in patients of multiple cancer types [24]. Furthermore, FGFR2 amplification has been shown to be accompanied with C-terminal exon abrogation, which regulates receptor internalization [25]. Impaired termination of FGFR signaling leads to continuous receptor activation [22]. In addition, mutation of FGFR can also render it insensitive to endocytosis by maintaining its expression at the cell surface [22]. A number of germline activating point mutations of FGFRs have been identified in human cancers and are associated with poor survival and chemoresistance [26, 27]. Mutations in the extracellular domain of FGFRs facilitate ligand binding, while mutations in transmembrane and kinase domains lead to constitutive activation of receptors (Figure 2A) [22]. Furthermore, alternative splicing of the third Ig-like domain could promote tumorigenesis. Paracrine signaling typically occurs through FGFR-IIIb that is expressed on mesenchymal cells and -IIIc expressed on epithelial cells [22]. In cancer models, however, the switch from FGFR IIIb to FGFR IIIc by alternative splicing results in autocrine activation of the receptor (Figure 2A) [28]. For example, FGFR2-IIIb to IIIc switch is related to increased invasiveness in bladder and prostate cancers [29, 30]. In addition, FGFR1-IIIc has been upregulated in pancreatic cancer where it is regarded as a strong oncogene [31].

### FGF2 as pro-angiogenic agent

FGF2 is an extremely potent pro-angiogenic growth factor. FGF2 exerts its effects on endothelial cells *via* a paracrine mode after being released by tumor and stromal cells or through mobilization from ECM (Figure 2B) [32]. In addition, FGF2 plays autocrine roles in endothelial cells [32]. It has been reported that endothelial cells predominantly express FGFR1 and to some extent FGFR2 [33, 34]. Activation of these receptors by FGF2 leads to endothelial cell proliferation, migration, protease production, and angiogenesis. Furthermore, the full mitogenic and chemotactic responses of FGF2 in endothelial cells require activation of ERK1/2 and protein kinase C (PKC) signaling pathways [35]. FGF2 upregulates plasmin-plasminogen activator (uPA) and matrix metalloproteinase (MMP) production in endothelial cells eventually leading to ECM degradation and angiogenesis [36]. In addition, the response of endothelial cells to FGF2 can be regulated by integrins [21]. Immobilized FGF2 binds to αvβ3 integrin causing endothelial cell adhesion, migration, proliferation, and morphogenesis (Figure 2B) [37]. There is also considerable cross-talk between FGF and vascular endothelial growth factor (VEGF) signaling, whereby FGF-induced signaling promotes resistance to VEGF receptor signaling for blocking of the VEGF [38]. Moreover, transient expression of FGF2 in endothelial cells control the expression of genes implicated in cell cycle, differentiation, adhesion, and cell survival [39]. Taken together, these data suggest an important role of FGF2 in promoting endothelial cell angiogenesis (Figure 2B).

### FGF2 as mitogen for tumor cells

Although FGF2 levels are elevated in several human cancers, FGF2 levels do not always correlate with microvessel density [40]. For example, in a study conducted by Kuwahara et al, majority of pancreatic ductal carcinomas were positive for VEGF and FGF2 [41]. A significant correlation was observed between VEGF expression and MVD but not between FGF2 and MVD [41]. However, in a study conducted by Garcia de la Torre et al, FGF2 expression was high in primary parathyroid hyperplasia (PPH) and FGF2 scores and MVD were significantly correlated [42]. Therefore, FGF2 may contribute to cancer progression through alternative mechanisms involve acting directly on tumor cells [19]. Mutations in genes encoding FGFs and FGFRs deregulate FGFR signaling [43, 44]. However, no activating mutations have been reported as yet for FGF2 [44]. FGF2-induced activation of FGFR signaling and subsequent activation of PI3K/Akt and ERK1/2 signaling pathways in cancer cells [19, 45]. FGF2 contributes to tumor progression through enhanced expression and/or release from tumor, endothelial, or stromal cells as well as release from local reservoirs in the ECM (Figure 2B) [43]. Secretion of proteases leads to release of the sequestered FGF2 [43]. Therefore, FGF2 functions in an autocrine or paracrine manner in cancer cells (Figure 2B).

### FGF2 as mitogen for stromal cells

Tumor progression to metastatic stage is promoted by fibroblasts in tumor stroma through secretion of multiple paracrine factors [46, 47]. FGF2 secreted by stromal fibroblasts induces tumor cell proliferation *via* FGFR paracrine signaling (Figures 2A, 2B) [48]. In addition, fibroblasts within tumor stroma can be activated by FGF2 secreted from endothelial and tumor cells (Figure 2B) [49]. Activated fibroblasts also produce proteases, such as MMPs that degrade ECM and promote secretion of growth factors including FGF2 in the tumor microenvironment [50].

### Dysregulated downstream FGF2/FGFR signaling pathway in hematological tumors

Growing evidence supports a role of FGF2 in hematopoiesis starting at early stages of development through adulthood. In early stages of development, FGF2 has an important role in the proliferation of hemangioblasts, which are common progenitors of hematopoietic and endothelial cells [51] that play a central role in hematopoietic and angiogenic differentiation [52]. In addition, FGF2 plays a role in self-renewal, cell survival, and cell adhesion of human embryonic stem cells [53]. In adult hematopoiesis, FGF2 induces proliferation of stromal cells of bone marrow [54]. FGF2 also induces the production of interleukin-6 (IL-6) [55] and counteracts the suppressive effect of transforming growth factor beta (TGF-β) on myeloid progenitor cells [56]. Myeloid precursor cells can be induced by FGF2 to give rise to erythroid progenitors [57]. In the absence of FGF2, myeloid progenitors generate cells of the neutrophil-granulocyte lineage upon FGF2 induction [58].

Neoplastic cells that define each hematological tumor are descendants of a specific lineage of the hematopoietic process. The involvement of FGF2 in various stages of hematopoiesis suggests that its dysregulation can result in hematological cancers [59]. FGFRs are expressed on all cell types of haematopoietic origin, and deregulation of FGFR gene expression or mutation has been observed in haematologic malignancies [59]. The ability of FGF2 to induce stem cell proliferation and differentiation implies that FGF2 is involved in very early stages of hematopoiesis. It has been reported that lymphoma cell lines express FGF2 and FGFRs and release FGF2 into culture media [45].

### Putative mechanisms of FGF2 in Hodgkin Lymphoma

Hodgkin lymphoma (HL) is a rare B-cell malignant neoplasm characterized by a paucity of malignant Hodgkin and Reed-Sternberg cells (HRS cells) embedded within an inflammatory infiltrate [60]. FGF2 causes aberrant signaling activities in HRS cells involving SDC1, NF-κB, IL-6, Ras/ERK, and JAK/STAT as shown below.

SDC1 regulates bioavailability, dimerization, and interaction of FGF2 with FGFRs [20]. Witzig and colleagues reported that SDC1 was expressed in the bone marrow of patients with plasma cell proliferative disorders [61]. In line with this, increased expression of FGF2 and SDC1 was also reported in HL cell lines [68]. Overall, findings suggest that increased expression of FGF2, FGFR, and SCD1 is associated with poor prognosis and chemoresistance [62].

**Figure 3:**
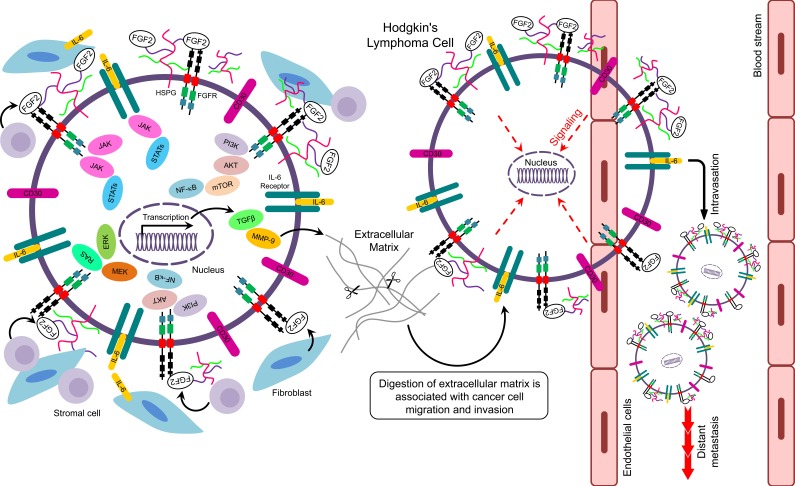
Putative signaling pathways related to FGF2 in Hodgkin's lymphoma

Activation of the NF-κB pathway is a well-established mechanism for protection of tumor cells from apoptosis [63, 64]. In HL, NF-κB is constitutively activated and represents an important step for the proliferation of neoplastic HRS cells (Figure 3) [65]. Epstein-Barr Virus (EBV) is a risk factor for HL [74]. In EBV-positive (EBV+) HL, the EBV oncoprotein, latent membrane protein-1 (LMP1), has been implicated in the activation of NF-κB signaling leading to enhanced B-cell survival [65]. Alternatively, NF-κB may serve as a transcription factor for the FGF2 gene regulating expression and release of FGF2 by LMP1 [66, 67]. This process could be of clinical importance for determining the relationship between EBV status and FGF2 levels in HL patients (Figure 3). IL-1β, which is expressed in subsets of cells in the HL tumor microenvironment, activates PI3K signaling pathway to upregulate FGF2 production through NF-κB [67].

The interleukin-6 (IL-6) signaling pathway has also been implicated in tumor progression [68, 69]. In multiple myeloma, stromal-derived IL-6 stimulates FGF2 expression in tumor cells, which in turn stimulates the secretion of IL-6 [70]. In addition, IL-6 and FGF2 together can enhance proliferation of myeloma cells [71]. In HL, IL-6 and its receptors are expressed by HRS cells (Figure 3) [72, 73]. Moreover, IL-6 is upregulated in serum of HL patients resulting in poor prognosis [74]. FGF2 upregulates IL-6 gene expression in the fibroblast NIH-3T3 cell line [75]. Moreover, in a basal cell carcinoma cell line, IL-6 mediates upregulation of FGF2 through activation of JAK/STAT3 and PI3K/Akt pathways which are aberrantly activated in HL [65]. Therefore, IL-6 and FGF2 may be involved in paracrine and autocrine interactions to promote chemoresistance in relapsed/refractory HL.

Components of the Ras/ERK pathway are aberrantly expressed in malignancies and associated with chemoresistance [76]. MEK/ERK signaling pathway is essential for proliferation and survival of neoplastic HRS cells (Figure 3) [77]. FGF2 induces MEK signaling to upregulate anti-apoptotic proteins and enhance chemoresistance [78]. In addition, FGF2 mediates chemoresistance to doxorubicin in endothelial cells by inhibiting the pro-apoptotic protein ASK1, which is a member of the MEKK family [79].

The Janus kinase-signal transducer and activator of transcription (JAK/STAT) is a frequently altered pathway in the pathogenesis of HL [80]. The JAK/STAT pathway has been implicated in FGF2-induced chemoresistance in human cancer cells [81]. The JAK2 inhibitor lestaurtinib can overcome JAK/STAT-induced drug resistance in refractory HL cell lines [82]. Therefore, FGF2 may promote chemoresistance by deregulation of JAK/STAT signaling in HRS cells of relapsed and refractory HL patients (Figure 3).

The above studies demonstrated key downstream signaling pathways in HL, including cell growth, survival, and angiogenesis. It seems clear that the main pathways contributing to HL are regulated, at least in part, by FGF2 (Figure 3). Therefore, clinical trials using agents that target FGF pathway may be promising for treatment of HL.

## CLINICAL PROGNOSTIC VALUE AND FUNCTIONAL SIGNIFICANCE OF FGF2 IN SOLID AND HEMATOLOGICAL TUMORS

Several studies have compared FGF2 serum levels in cancer patients to those in healthy volunteers (Tables 2 and 3). FGF2 expression in sera quantified by ELISA was strongly increased in cancer patients compared to healthy donors. Significant correlations between serum FGF2 levels and tumor stage, size, and metastasis were reported in endometrial, colorectal, esophageal, head and neck, liver, renal, and testicular cancers. However, no significant correlation was observed between increased serum FGF2 levels and tumor grade in bladder, breast, lung, and prostate cancers. In all leukemia and lymphoma studies, there was no correlation between increased serum levels of FGF2 and microvessel density or stage of the disease. However, high serum FGF2 levels were significantly correlated with tumor bulkiness in Non-Hodgkin's lymphoma (NHL). Therefore, the levels of serum FGF2 may have prognostic significance in these cancers, and quantification of FGF2 may provide an indirect, non-invasive way to monitor patients with high risk of relapse from solid and hematological tumors (Tables 2 and 3).

**Table 2:** Studies evaluating FGF2 as a prognostic biomarker in cancer patients with solid tumors

Cancer Type	Patient Number	Specimen Type	Cancer Subtype	Method	FGF2 Expression Pattern	Prognosis	Associated with	Ref
Bladder Cancer	32	Resection	−	RT-PCR	Elevated in high stage vs. low stage patients (p= 0.001)	−	High stage, local relapse	[129]
	82 vs. 20 controls	Untreated serum	Noninvasive, invasive	ELISA	(↑) FGF2 levels vs. controls (p=0.083); (↑) FGF2 in noninvasive vs. invasive (P= 0.013)	No significant difference	No correlation with tumor grade, patient age	[130]
Breast Cancer	64	Cytosolic extract	Primary	ELISA	(↑) >2 fold FGF2 levels in tumor vs. controls and non-malignant mastectomy specimens (p < 0.01)	−	−	[131]
	79	Sections	−	IHC	(↑) FGF2 in 38% neoplastic cells, 37% in stromal cells	−	Recurrence, aggressiveness	[132]
	136 vs. 65 controls	Diagnostic biopsy,	ER-(+) and (−); PR (+) and (−)	IHC	In 84% tumors FGF2 staining limited to cytoplasm, 16% tumors limited to both cytoplasm and nuclei vs. positivity limited to the cell nuclei of the basal layer of mammary ducts in normal mammary epithelium	−	Not correlated with clinical, pathological and biological characteristics	[133]
		Serum		ELISA	(↑) in tumors vs. healthy controls (p < 0.001)			
	111	Treated resection	ER (+/−)- PR (+/−)	IHC	70% tumors positive, 30% tumors showed strong staining, (↑) with histological grade (p < 0.05)	(↓) OS in FGF2 (+)vs. (−) group in negative nodal status sub-group	Negatively correlated with histological grading (p < 0.05)	[134]
	149, 14 non-neoplastic, 7 controls	Resection	Primary	ELISA	(↑) ~3 fold FGF2 in tumors vs. non-neoplastic tissues (p< 0.0001); (↑) ~12 fold FGF2 in tumors vs. normal control tissues (p= 0.0003)	No significant difference	No correlation between FGF2 and MVD	[135]
	97 vs. 46 controls	Untreated nipple aspirate	DCIS and invasive	ELISA	(↑) 11 fold FGF2 in cancer patients vs. controls (p < 0.0001)	−	No correlation with tumor stage, size, nodal spread	[136]
	148	Untreated resection	Triple negative	IHC	13% tumors positive vs. normal breast tissue	No significant difference	Basal type cancer	[137]
Colorectal Cancer	124, 26 polyp patients, vs. 55 controls	Plasma	Primary	ELISA	(↑) 1.8 fold in tumor vs. normal controls (p=0.0004), (↓) 0.6 fold in disease-free patients at follow up vs. pre-operative (p= 0.0004)	−	Metastasis	[138]
	35 obstructing carcinoma, 34 non-obstructing	Untreated resection	Obstructing and non-obstructing	IHC	(↑) 1.7 fold FGF2+ inflammatory cell in obstructing vs. non-obstructing carcinoma (p=0.018); no difference in FGF2 between obstructing vs. non-obstructing carcinoma	−	Hsp47 and stromal myofibroblast fibrosis	[139]
Endometrial Cancer	134 (type I=70 and type II=64) vs. 64 controls	Untreated serum	Type I and II	ELISA	(↑) ~10 and 20 fold FGF2 in type I and type II, respectively vs. healthy controls	(↓) OS and DFS: in type I with high vs. low FGF2 levels and type II with high vs. low FGF2 levels	Advanced tumor stages	[140]
Esophageal Cancer	70 vs. 20 controls	Untreated, treated tissues	ESCC	IHC, WB	Positive expression in tumors vs. absent in normal tissues	(↓) 0.3 fold 2 yr RFS FGF2 expression ((P = 0.005)	Local recurrence, reduced RFS	[141]
Glioma	21	Untreated resection	Astrocytoma, Glioblastoma	IHC	87% tumors positive for FGF2 expression vs. absent in normal tissues	−	Degree of malignancy	[142]
	61	Resection	Astrocytoma	IHC	44% tumors had strong FGF2 expression, stronger staining in higher grades than lower grades (p < 0.05)	(↓) survival in tumors with strong vs. weak staining	High grade tumors	[143]
Head and Neck	66 vs. 18 controls	Resection	SCC	IHC, ELISA	(↑) ~ 12 fold FGF2 expression in tumors vs. control (P < or = 0.05)	−	Early stage disease	[144]
Liver Cancer	88	Untreated serum	−	IHC and ELISA	(↑) high serum FGF2 levels	(↓) 0.5 fold months DFS in patients with high (>10.8 pg/mL ) vs. low (<10.8 pg/mL) FGF2 level	Tumor size, invasion, advanced stage, platelet count	[145]
	16	Untreated resection	−	IHC, RT-PCR, WB	Positive expression in hepatoma vs. absent in non-cancerous liver cells	−	−	[146]
Lung Cancer	106 vs. 17 controls	Serum	ACA, SCC, SCLC	ELISA	(↑) 2.5 fold serum FGF2 levels in tumors vs. normal controls (p< 0.05)	(↑) OS in SCLC patients with high (> 5.4 pg/ml) vs. low (< 5.4 pg/ml) FGF2	−	[147]
	103	Untreated serum	SCLC	ELISA	25% patients had FGF2 ≥17 pg/ml	(↓) 7.5 and 0.5 fold 1 yr, 2 yr survival in high ( ≥17 pg/ml) vs. low ( < 17 pg/ml) FGF2 (p = 0.0026)	Poor OS	[148]
	184 vs. 100 controls	Untreated serum	SCLC, NSCLC	ELISA	(↑) FGF2 levels NSCLC median 4.2 pg/ml; SCLC median 1.8 pg/ml;	(↓) ~0.5 mo. survival in high (>3.4 pg/ml) vs. low (<3.4 pg/ml) FGF2 (p= 0.023)	No correlation with clinicopathological parameters	[149]
	40 vs. 22 controls	Untreated serum	NSCLC	ELISA	(↑) 1.6 fold FGF2 levels in tumors vs. controls (p=0.01)	(↓) ~0.5 mo. survival in high (>11.21 pg/ml) vs. low (<11.21 pg/ml) FGF2	No correlation with stage, TSP-2 concentration	[150]
Melanoma	76, 43 nevi, 10 dysplastic controls	Biopsy	NM, SSM	IHC	Strong cytoplasmic expression in malignant vs. nuclear staining in benign nevi.	−	Stromal localization	[151]
Oral Squamous Cell Carcinoma	61	Untreated biopsy	SCC	IHC	Positive FGF2 expression in cancer cells	(↓) ~0.5 fold survival in FGF2 (+) vs. (−) expression (p < 0.01)	Poor differentiation, mode of invasion, lymph node metastasis	[152]
Osteosarcoma	80	Surgical and biopsy	Intramedullary	IHC	57.5% tumors strong positive with cytoplasmic and epithelium FGF2 expression	(↓) ~0.5 fold mo. OS in (+) vs. (−) FGF2 (p< 0.006)	MVD	[153]
Ovarian Cancer	117 tumors; 54 benign, 42 normal ovaries	Untreated serum	−	Fluorokine MAP multiplex kits	(↑) 1.6 fold FGF2 levels in malignant tumors vs. controls	No significant difference	PDGF-AA (p<0.001)	[154]
	39 vs. 11 controls	Untreated surgical, serum	Serous endometrioid clear cell mixed Braner	RT-PCR	FGF2 gene expression strong in malignant vs. weak detection in control	_	_	[155]
				IHC	Strong positive in tumors vs. weak staining in normal control			
				ELISA	(↑) in tumors vs. controls (p=0.04)			
Pancreatic Cancer	78 vs. 16 controls	Surgical	−	Northern blot	(↑) 9-fold in tumors vs. normal controls (p<0.01)	(↓) ~ 0.6 fold months OS in positive vs. negative FGF2 (P < 0.001)	Advanced tumor stage	[156]
				IHC	Intense staining in cytoplasm and nucleus of cancer cells vs. weak cytoplasmic staining in normal controls			
	46	TMA blocks	Ductal ACA	IF	Cytoplasmic FGF2 expression in tumors vs. nuclear expression in myofibroblasts; (↓) nuclear FGF2 staining in tumors vs. positive in stromal cells (35%) (P < 0.0001)	_	_	[157]
Prostate Cancer	55 vs. 32 benign controls	Untreated serum	−	ELISA	(↑) 2-fold FGF2 in tumors vs. control (P < 0.0007)	−	No correlation with clinical stage, Gleason grade	[158]
	47 (36 patients + 11 benign) vs. 23 controls	Serum	−	ELISA	(↑) 5 fold FGF2 levels in tumors vs. control (P= 0.0002)	−	High PSA levels (>100 ng/ml)	[159]
		Tissue sections		IHC	strong cytoplasmic expression in carcinoma cells vs. negative benign epithelia			
	31 vs. 11 controls	Resection	−	ELISA	(↑) 2.5 fold FGF2 levels in tumors vs. control (P < 0.005)	_	No correlation with Gleason score, pathological stage	[160]
				IHC	Strong stromal and endothelial staining (not epithelial)			
Renal Cancer	206 vs. 10 benign controls	Untreated serum	−	ELISA	(↑) >3 fold FGF2 in tumors vs. benign controls (P=0.03)	(↓) OS in high (>3.0 pg/ml) vs. low FGF2 (<3.0pg/ml)	Tumor stage, tumor grade	[161]
Testicular Cancer	21 vs. 22 control	Serum, tumor biopsy	−	ELISA	(↑) ~ 7.3 fold serum FGF2 levels in tumors vs. control (P < 0.001), positive expression in all tumor biopsies	−	Tumor stage	[162]
Thyroid Cancer	35 vs. 26 controls	Untreated serum	Papillary carcinomas	ELISA	(↑) ~ 2 fold serum FGF2 levels in tumors vs. controls (p < 0.05)	−	−	[163]

**Table 3:** Studies evaluating FGF2 as a prognostic biomarker in cancer patients with hematological tumors

Cancer Type	Patient Sample Number	Specimen Type	Method	FGF2 Expression Pattern	Prognosis/Associated with	Ref
Acute Lymphoblastic Leukemia (ALL)	28 vs. 11 controls	Untreated, treated plasma	ELISA	(↑) ~1.4 fold plasma FGF2 levels in tumors vs. normal controls	−	[164]
	22 pediatric patients vs. 39 controls	Untreated urine	ELISA	(↑) ~8 fold urine FGF2 levels in tumors vs. normal controls (p< 0.0001)	−	[165]
Acute Myeloid Leukemia (AML)	113 vs. 11 controls	Untreated, treated plasma	ELISA	(↑) 1.2 fold plasma FGF2 levels in tumors vs. normal controls	−	[164]
	81 vs. 18 controls	Untreated BM biopsy	IHC	(↑) 1.6 fold FGF2 levels in tumors vs. normal controls (p=0.04)	No significant correlation between FGF2 and MVD	[166]
Chronic Lymphocytic Leukemia (CLL)	39 vs. 11 controls	Treated, untreated peripheral blood- plasma	ELISA	(↑) FGF2 levels in 54% tumors vs. normal range in healthy controls	−	[167]
	155 vs. 11 controls	Untreated, treated plasma	ELISA	(↑) ~9 fold plasma FGF2 levels in tumors vs. normal controls	−	[164]
	14 vs. 58 controls	Urine	ELISA	(↑) 2 fold FGF2 levels in tumors vs. controls ( P= 0.0001)	−	[168]
	36 vs. 15 controls	Peripheral blood (cell lysates and plasma)	ELISA	(↑) ~64 fold FGF2 levels in tumors with high risk vs. normal controls ( P< 0.0001)	No significant correlation between FGF2 and factors other than stage of disease	[169]
Chronic Myelogenous Leukemia (CML)	16 vs. 11 controls	Treated, untreated peripheral blood- plasma	ELISA	(↑) FGF2 levels in 44% tumors vs. normal range in healthy controls	−	[167]
	53 vs. 11 controls	Untreated, treated plasma	ELISA	(↑) 1.6 fold plasma FGF2 levels in tumors vs. normal controls	−	[164]
Hairy Cell Leukemia (HCL)	7 vs. 7 controls	Treated, untreated serum and BM aspirates	ELISA	Serum- (↑) ~37 fold FGF2 levels in tumors vs. absent in controls (p< 0.05); BM aspirate: (↑) 16 fold FGF2 levels in tumors vs. absent in controls (p< 0.001)	−	[170]
Hodgkin's Lymphoma	39	Lymph nodes tissue	IHC	85% tumors positive	−	[73]
	67	TMA (NS)	RT-PCR	(↑) 246 fold FGF2 levels in PO tumors vs. normal lymph node controls (↑) 10 fold FGF2 levels in GO tumors vs. normal lymph node controls	−	[62]
			IHC	Strong positive staining in PO patients than GO patients		
	37	Untreated, treated serum	ELISA	FGF2 levels in tumors were normal (p= 0.075)	No significant change in FGF2 levels relative to pre-therapy values	[171]
Multiple Myeloma	18 vs. 4 controls	BM aspirates	RT-PCR	(↑) FGF2 expression in tumors (13.5 pg/mL) vs. absent in controls (P= 0.02)	−	[70]
	44 and 12 anemia patients	Plasma cells	ELISA	(↑) 6.7 fold FGF2 levels in active MM patients vs. non-active ones (p < 0.01)	No significant correlation between FGF2 and BM neovascularization	[172]
	56 vs. 20 controls	Untreated, treated serum	ELISA	(↑) FGF2 levels in tumors vs. controls (p < 0.001) (↓) 0.3 fold FGF2 levels in treated patients with CR vs. untreated patients (p<0.001)	Significant correlation between FGF2, VEGF, HGF, and B2M	[173]
Non-Hodgkin's Lymphoma	58 untreated, 19 treated, 11 controls	Untreated, treated serum	ELISA	(↑)~2 fold serum FGF2 levels in tumors vs. controls (p < 0.001)- No correlation between FGF2 at diagnosis and after treatment	Correlated with bulky disease	[174]
	39	Biopsy	IHC	Positive expression in 23.1% tumors	(↓) 0.5 and 0.4 fold months OS (p=0.033) and PFS (p=0.003), respectively, in FGF2 positive vs. negative tumors; -correlated with bulky disease	[175]
	27	BM biopsy	IHC	7 % positive FGF2 in tumors	−	[176]
	65	Untreated, treated serum	ELISA	(↑) Untreated FGF2 in tumors vs. controls (p< 0.001) - no significant change in FGF2 relative to untreated sample values	−	[171]

BM: bone marrow; B2M: Beta-2 microglobulin; CR: complete remission; ELISA: the enzyme-linked immunosorbent assay; GO: good outcome; HGF- hepatocyte growth factor; IHC- immunohistochemistry; MM: multiple myeloma; MVD: microvessel density; NHL- Non-Hodgkin Lymphoma; NS: nodular sclerosis; OS: overall survival; PFS: progression free survival; Poor outcome; TMA: tissue microarray; VEGF: vascular endothelial growth factor.

FGF2 expression in cancer surgical sections has been evaluated using immunohistochemical, Western blot, and qRT-PCR techniques (Tables 2 and 3). Up-to-date diagnostics and antibodies that allow detection and precise quantification of FGF2 are listed in Table 4. Immunohistochemical and immunofluorescence studies have shown that FGF2 staining is heterogeneous and significantly increased in malignant tissues compared to benign or normal tissues (Tables 2 and 3). The differential expression and localization of FGF2 was also studied in different cancers. For example, FGF2 expression is generally limited to the cytoplasm of breast cancer tissues, while it is exclusively expressed in the nuclei of normal mammary tissues. Similarly, FGF2 is strongly expressed in the cytoplasm of malignant melanocytes and prostate cancer tissues, while it is almost entirely restricted to the nuclei of benign cells. In pancreatic cancer cells, FGF2 staining is intense in both the cytoplasm and nucleus, while it is weak in normal control tissues. Different expression and localization of FGF2 suggests that FGF2 and different members of FGFR may have different functions and signaling in various cancers. Moreover, FGF2 expression was elevated in tumor stroma, including inflammatory cells, myofibroblasts, and endothelial cells in colorectal, pancreatic, and prostate carcinomas, respectively (Table 2). These findings suggest that FGF2 can modulate tumor progression by activating signaling pathways in cancer-associated fibroblasts, endothelial cells, and cancer cells. In addition, fibroblasts that are abundant in the stroma of carcinomas at advanced stages of disease can mediate resistance to treatment *via* FGF2 secretion [83]. Additional studies on tissue sections have revealed that high FGF2 intratumoral levels are associated with advanced tumor stages of bladder, glioma, head and neck, liver, and prostate cancers (Table 2).

**Table 4:** *In vitro* diagnostics (IVD) and research use only (RUO) detection methods for FGF2

Diagnostic Type	Source (host)	Reactivity	Manufacturer	Market Status	Applications	Ref
Antibody (Clone FGF288)	Mouse	Unique synthetic peptide of FGF2 coupled to keyhole limpet hemocyanin	Biogenex (CA, USA)	Class I IVD	IHC	*
Antibody (Clone 3D9)	Mouse	Recombinant fragment corresponding to amino acids 10-155 human FGF2	Novus Biologicals (CO, USA); Enzo Life Sciences (NY, USA)	RUO	WB, IF, IHC	**
Antibody (Clone 10043)	Mouse	Biotin conjugated; detects human FGF2 in ELISA	Novus Biologicals (CO, USA); R&D Systems (MN, USA)	RUO	ELISA	[177]
Antibody (Clone 10060)	Mouse	Recognizes human FGF2	Novus Biologicals (CO, USA)	RUO	ELISA	[177, 178]
Antibody (Clone 2H5G2C1)	Mouse	Purified recombinant fragment of human FGF2	Thermo Scientific (MA, USA); Sigma-Aldrich (MO, USA)	RUO	WB, IHC	**
Antibody (Clone FB-8)	Mouse	Recombinant full length human FGF2	Novus Biologicals (CO, USA); Thermo Scientific (MA, USA)	RUO	WB, ELISA, RIA	[179]
Antibody (Clone MC-GF1)	Mouse	Recombinant full length human FGF2	Novus Biologicals (CO, USA); LifeSpan Biosciences (WA, USA)	RUO	WB, ELISA, IHC	[180]
Antibody (Clone AS24)	Mouse	Human FGF2	Santa Cruz (TX, USA); Thermo Scientific (MA, USA)	RUO	IHC, ELISA	**
Antibody (Clone AS25)	Mouse	Biotin Conjugated; Human FGF2	Abcam (UK); Santa Cruz (TX, USA)	RUO	ELISA	**
Antibody (Clone F-343)	Mouse	Full length native human FGF2	Abcam (UK); Thermo Scientific (MA, USA)	RUO	ELISA, WB	**
Antibody (Clone F-474)	Mouse	Full length native human FGF2	Abnova (Taiwan); Abcam (UK)	RUO	ELISA	[181]
Antibody (Clone F-74)	Mouse	Full length native human FGF2	Abnova (Taiwan); Abcam (UK)	RUO	ELISA	**
Antibody (Clone JKFb-1)	Mouse	Recombinant human FGF2	Novus Biologicals (CO, USA);	RUO	ELISA	**
Antibody (Clone bFM-1)	Mouse	Purified bovine brain FGF2	EMD Millipore (MA, USA)	RUO	RIA	[182, 183]
Antibody (Clone bFM-2)	Mouse	Purified bovine brain FGF2	EMD Millipore (MA, USA)	RUO	WB, IHC, RIA	[184, 185]
Antibody (Clone 17F4.1)	Mouse	Recombinant human FGF2	EMD Millipore (MA, USA)	RUO	Neutralizing	**
Antibody (Clone EP1735)	Rabbit	Synthetic human FGF2	Abcam (UK); OriGene (MD, USA)	RUO	WB, IP, FCM, ELISA	**
Antibody (Clone MM0276-6D38)	Mouse	Recombinant human FGF2	Novus Biologicals (CO, USA); Abcam (UK)	RUO	WB	**
Antibody (Clone NYR-hFGF-b)	Mouse	Full length human FGF2	Abcam (UK)	RUO	WB, IP, ELISA	**
Antibody (Clone 0.T.50)	Mouse	Full length native bovine brain FGF2	Abcam (UK)	RUO	Neutralizing	[186]

* information taken by contacting company's technical department

** information taken from company's website ELISA: enzyme-linked immunosorbent assay; FCM: flow cytometry; IF: immunofluorescence; IHC: immunohistochemistry; IP: immunoprecipitation; IVD: in vitro diagnostic; RIA: radioimmunoassay; RUO: research use only; WB: Western blot

There is an urgent need for the identification of novel prognostic biomarkers to improve treatment of poor outcome cancer patients. It is worth noting that in the vast majority of studies, high serum and intratumoral FGF2 levels were associated with reduced cancer patient survival. In addition, high intratumoral and serum levels of FGF2 were associated with relapse and/or recurrence in various cancers such as bladder, breast, esophageal cancers and HL (Tables 2 and 3). In spite of a few contradictory finding, FGF2 is considered a significant tumor biomarker and a potential therapeutic target. Ongoing and future clinical trials are warranted to determine whether FGF2 could be incorporated in cancer prognosis and whether FGF targeting therapies have a favorable effect on cancer recurrence and mortality.

## TARGETING THE FGF2/FGFR PATHWAY IN CANCER

**Figure 4:**
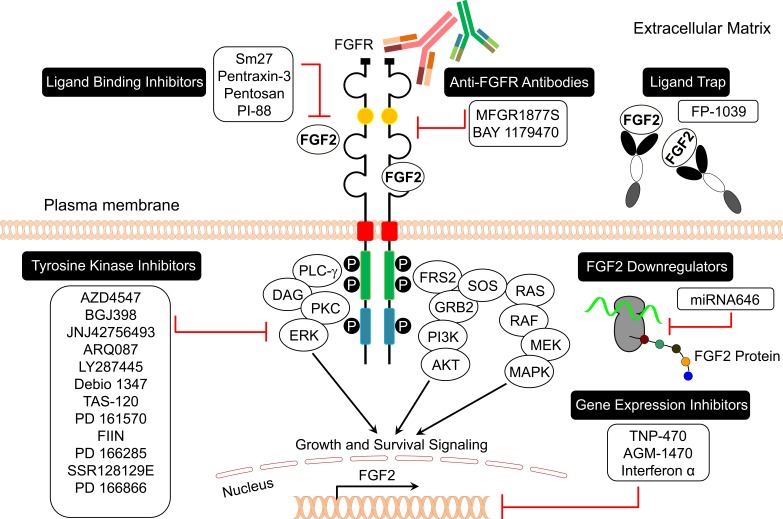
FGF2/FGFR signaling inhibitors in cancer

The trend in cancer personalized medicine is to search for biomarkers to predict a patient's response to the targeted therapy and the emergence of secondary resistance [84]. High expression of FGF2 correlates with a worse survival for relapsed/refractory cancer patients (Tables 2 and 3). Therefore, targeting FGF signaling may provide opportunities for personalized therapy in those patients. Several FGF2/FGFR inhibitors have shown promising anticancer and antiangiogenic efficacy in several *in vitro* assays and *in vivo* preclinical animal models (Table 5, Figure 4). Clinical studies on these compounds have been conducted in the last decade to evaluate their safety, efficacy, and tolerability (Table 6). Ongoing clinical trials are recruiting patients with metastatic, advanced, or relapsed/refractory cancers to evaluate the importance of blocking the FGF2/FGFR signaling in progressive and poor outcome cancer patients (Table 6).

**Table 5:** Agents target FGF2/FGFR in cancer

Agent	Company	Target	Agent Type	Characteristics	Clinical Trial	Indication/Tested on	Ref.
FGF2 inhibitors							
FP-1039/GSK3052230	Five Prime Pharmaceuticals (CA, USA)	FGF2	Protein	Ligand trap: prevents FGF2 from binding to receptors	Phase I (Ongoing) NCT01868022	Squamous non-small cell lung cancer, mesothelioma	[86]
Interferon-α	−	FGF2	Protein	Inhibits FGF2 expression and production	Phase II (ongoing) NCT00049530	Bladder cancer, melanoma	[87, 187]
miRNA 646	−	FGF2	miRNA	Downregulates FGF2	−	Osteosarcoma	[188]
Sm27	−	FGF2	Small molecule	Binds to heparin-binding site on FGF2 and prevents FGF2 interaction with receptors	−	Endothelial cells	[189]
Anvirzel	Nerium Biotechnology (Canada)	FGF2	Glycoside	Inhibits FGF2 export by affecting Na+/K+ pump	−	Prostate cancer	[190]
Pentraxin-3	−	FGF2	Protein	Inhibits FGF2 binding to FGFR	−	Pancreatic cancer	[191]
TNP-470/AGM-1470	−	FGF2	Antibiotic	Suppresses expression and production of FGF2	−	Bladder cancer	[192]
Pentosan Polysulfate (Elmiron)	Ortho-McNeil Pharmaceutical (NJ, USA)	FGF2	Small molecule	Blocks and inhibits activity of FGF2	−	Various advanced cancers	[193]
PI-88	Progen Pharmaceuticals (Australia)	FGF2	Small molecule	Binds and inhibits FGF2 associated angiogenesis	−	Liver cancer	[194]
Thalidomide	Celgene (NJ, USA)	FGF2	Small molecule	Inhibits FGF2 induced angiogenesis	−	Multiple cancers	[195]
PAMPS, PAS, PSS, PVS	−	FGF2	Sulfonic acid polymers	FGF2 Antagonists	−	Endothelial cells	[196]
Sirolimus (Rapamycin)	Pfizer (NY, USA)	FGF2	Small molecule	Inhibits FGF2 dependent angiogenesis and proliferation	−	Fibroblasts and endothelial cells	[197]
Suramin (Germanin)	Bayer (Germany)	FGF2	Small Molecule	FGF2 antagonist/reduced FGF2 expression	−	Multiple cancers	[198]
Platelet Factor 4	−	FGF2	Protein	FGF2 antagonist	−	Endothelial cells	[199]
Non-specific Tyrosine Kinase Inhibitors							
Lenvatinib (Lenvima)	Eisai (Japan)	FGFR1, PDGFR, VEGFR	Small molecule	Tyrosine kinase and angiogenesis inhibitor	Approved	Progressive, radioactive iodine-refractory thyroid cancer	[200]
AP 24534 (Ponatinib, Iclusig)	ARIAD Pharmaceuticals (MA, USA)	BCR-ABL, FGFR1-4	Small molecule	Tyrosine kinase inhibitor	Approved	CML, ALL	[89]
Pazopanib (Votrient)	GlaxoSmithKline (England)	FGFR, PDGFR, VEGFR	Small molecule	Tyrosine kinase and angiogenesis inhibitor	Approved	Renal cell carcinoma, soft tissue sarcoma	[90]
Nintedanib (Vargatef, Ofev)	Boehringer Ingelheim (Germany)	FGFR1-3, PDGFR, VEGFR	Small molecule	Tyrosine kinase and angiogenesis inhibitor	Approved (EU)	Non-small-cell lung cancer	[201]
BMS582664 (Brivanib)	Bristol-Myers Squibb (NY, USA)	FGFR1, VEGFR1, VEGFR2	Small molecule	Tyrosine kinase and angiogenesis inhibitor	Phase I/II/III trials NCT00633789 NCT00355238 NCT00435669	Liver cancer, solid tumors	[202]
SU6668, TSU-68 (Orantinib)	SUGEN/Pfizer/Taiho Pharmaceutical (CA, USA/NY, USA/Japan)	FGFR, PDGFR, VEGFR2	Small molecule	Tyrosine kinase and angiogenesis inhibitor	Phase I/II NCT00024206 NCT00784290	Advanced solid tumors, liver cancer	[203, 204]
TKI-258, CHIR-258 (Dovitinib)	Novartis (Switzerland)	FGFR1-3, PDGFR, VEGFR	Small molecule	Tyrosine kinase and angiogenesis inhibitor	Phase II trials NCT01058434 NCT01831726 NCT01861197 NCT01732107 NCT01719549	Multiple cancers including relapsed MM, non-small cell lung cancer, bladder cancer, gastric cancer	[205]
E3810 (Lucitanib)	EOS/Clovis Oncology (India/CO, USA)	FGFR1-3, VEGFR	Small molecule	Tyrosine kinase inhibitor	PhaseI/II NCT01283945	Solid tumors	[206]
Specific FGFR inhibitors							
Debio 1347	Debiopharm (Switzerland)	FGFR1-3	Small molecule	Inhibits FGFR autophosphorylation	Phase I NCT01948297	Advanced solid tumors	[97]
AZD 4547	AstraZeneca (England)	FGFR1-3	Small molecule	Tyrosine kinase inhibitor	Phase II NCT01795768	Gastric cancer, esophageal cancer, breast cancer	[98]
BGJ 398	Novartis (Switzerland)	FGFR1-3	Small molecule	Tyrosine kinase and angiogenesis inhibitor	Phase I NCT01004224	Advanced solid tumors	[207]
JNJ-42756493	Janssen Oncology (Belgium)	FGFR	Small molecule	Tyrosine kinase inhibitor	Phase I/II NCT01703481 NCT02365597	Urothelial cancer, glioma	[208]
ARQ 087	Arqule (MA, USA)	FGFR	Small molecule	Tyrosine kinase inhibitor	Phase I NCT01752920	Solid tumors	[209]
LY287445	LKT Laboratories (MN, USA)	FGFR1-4	Small molecule	Tyrosine kinase inhibitor	Phase I NCT01212107	Advanced tumors	[210]
TAS-120	Taiho Pharmaceuticals (Japan)	FGFR	Small molecule	Irreversible FGFR inhibitor	Phase I/II NCT02052778	Advanced solid tumors, multiple myeloma	[211]
MFGR1877S	Genentech/Roche (CA, USA/Switzerland)	FGFR3	Antibody	Inhibits FGFR3 mediated cell proliferation	Phase I NCT01363024 NCT01122875	Solid tumors, multiple myeloma	[212, 213]
BAY 1179470	Bayer (NJ, USA)	FGFR2	Antibody	Inhibits FGFR2 mediated cell proliferation	Phase I NCT01881217	Advanced, refractory solid tumors	[100]
PD 161570	Parke-Davis/Pfizer (NY, USA)	FGFR	Small molecule	Tyrosine kinase and receptor phosphorylation inhibitor	−	Ovarian cancer	[214]
PD 173074	Parke-Davis/Pfizer (NY, USA)	FGFR	Small molecule	Tyrosine kinase and angiogenesis inhibitor	−	Urothelial carcinoma	[215]
PD 166285 dihydrochloride	Parke-Davis/Pfizer (NY, USA)	FGFR	Small molecule	Tyrosine kinase and angiogenesis inhibitor	−	Small cell lung cancer	[215, 216]
PD 166866	Parke-Davis/Pfizer (NY, USA)	FGFR1	Small molecule	Tyrosine kinase and angiogenesis inhibitor	−	Small cell lung cancer	[217, 218]
FIIN hydrochloride	−	FGFR1-4	Small molecule	Irreversible FGFR inhibitor	−	Cancer cell lines	[219]
SU 5402	−	FGFR, VEGFR	Small molecule	Tyrosine kinase and angiogenesis inhibitor	−	Urothelial carcinoma	[215]
SSR128129E	−	FGFR	Small molecule	Binds extracellular domain. Inhibits FGFR signaling	−	Endothelial cells, cancer cell lines	[220, 221]

**Table 6:** Clinical trials related to FGF2/FGFR pathway

Clinical Trial Description ( Trial #)	Participants #	Start Date/Trial Status	Originator	Sponsor	Mechanism of Action	Study Type/Purpose
FGF2 Inhibitors						
Phase II study of low dose Pegintron (PEG interferon alfa-2b) in patients with metastatic melanoma over-expressing FGF2 (NCT00049530)	32	Sept 2003 / not recruiting - ongoing	Enzon Pharmaceuticals (Piscataway, NJ)	Eastern Cooperative Oncology Group (Boston, MA)	FGF2 inhibitor, interferon alpha stimulant	Interventional, response level of suppression of plasma FGF2 level with low dose Pegintron
Phase I, open-label, dose-finding study of FP-1039 in advanced solid tumors (NCT00687505)	39	July 2008 / completed	Five Prime Therapeutics, Inc. (San Francisco, CA)		FGFR1 inhibitor	Interventional, assess safety and tolerability
Non-Specific Tyrosine Kinase Inhibitors						
Phase I dose escalation study of Lenvima (Lenvatinib) in patients with solid tumors (NCT00280397)	27	Jan 2006 – Nov 2008 / completed	Eisai Inc. (Japan)		PDGFR-beta inhibitor; c-kit inhibitor; FGFR inhibitor; VEGFR 1-3 inhibitor	Interventional; adverse events, safety, tolerability
Phase Ib/II, open-label, multicenter study of Lenvima (lenvatinib) alone, and in combination with Everolimus in subjects with unresectable advanced or metastatic renal cell carcinoma following one prior VEGF-targeted treatment (NCT01136733)	180	Aug 2010 / not recruiting - ongoing	Eisai Co. Ltd. (Japan)		PDGFR-b antagonist; VEGFR-2 antagonist, FGFR inhibitor	Interventional, assess the dose-limiting and maximally tolerated toxicity, recommended dose, progression-free survival
Phase II, multicenter, randomized, open-label study of Votrient (Pazopanib) in thyroid carcinoma (NCT01813136)	168	Mar 2013 / ongoing - recruiting	Centre Leon Berard (France)	GlaxoSmithKline (Philadelphia, PA)	PDGFR antagonist; BRAF inhibitor; c-kit inhibitor; VEGFR 1-3 antagonist	Interventional, efficacy (objective response rate)
Phase I/II study of Orantinib for advanced hepatocellular carcinoma (NCT00784290)	35	Sept 2003 / completed	Pfizer (New York City, NY)	Taiho Pharmaceutical Co., Ltd. (Japan)	FGF inhibitor; PDGF inhibitor; VEGFR-2 antagonist	Interventional, assess the safety and response rate
Phase II study of Dovitinib in patients with gastrointestinal stromal tumors refractory and/or Intolerant to Imatinib (NCT01478373)	150	Jan 2012 - July 2014 / completed	Novartis (East Hanover, NJ)		FGF2 inhibitor; PDGFR Δ inhibitor; VEGFR inhibitor	Interventional, measure safety and efficacy
Phase II, open-label study of Lucitanib in patients with FGFR1-driven lung cancer (NCT02109016)	40	Apr 2014/ recruiting - ongoing	Advenchen Laboratories (Moorpark, CA)	Clovis Oncology, Inc. (Boulder, CO)	FGFR 1-3 inhibitor; VEGFR1-3 inhibitor	Interventional, efficacy (objective response rate)
Phase II study of Vargatef (Nintedanib) in patients with advanced FGFR3 mutated, overexpressed, or wild type urothelial carcinoma of urinary bladder (NCT02278978)	129	Oct 2014/not recruiting - ongoing	Boehringer Ingelheim (Germany)	National Taiwan University Hospital (Taiwan)	PDGFR α-Δ inhibitor; FGFR 1-3 inhibitor; VEGFR 1-3 inhibitor	Interventional, safety study with primary purpose of treatment
Phase I/II, multicenter, randomized, double-blind study of Vargatef (Nintedanib) in combination with paclitaxel for treatment of patients with BRAF wild-type metastatic melanoma (NCT02308553)	126	Jan 2015 / ongoing - recruiting	Boehringer Ingelheim (Ridgefield, CT)	Prof. Dr.Dirk Schadendorf (Germany)	PDGFR-alpha/beta inhibitor; FGFR1-3 inhibitor; VEGF1/2 inhibitor	Interventional, measure of progression-free survival, safety, tolerability
Phase III study to compare efficacy and safety of Masitinib in combination with Bortezomib and Dexamethasone to placebo in combination with Bortezomib and Dexamethasone in patients with relapsing multiple myeloma (NCT01470131)	300	Apr 2013	Masitinib: AB Science (France)	Masitinib: AB Science (France)	FGFR modulator; PDGFR antagonist	Interventional, assess overall time to progression and overall survival
			Bortezomib: Millennium Pharmaceuticals (Cambridge, MA)		Immunomodulator; proteasome inhibitor	
			Dexamethasone: Allergan (Ireland)		Glucocorticoid receptor agonist	
FGFR Inhibitors						
Phase I, multicenter, open label study of oral Debio 1347 (CH5183284) in patients with advanced solid malignancies, whose tumors have an alteration of the FGFR 1, 2 or 3 genes (NCT01948297)	112	Aug 2013 / ongoing - recruiting	Chugai Pharmaceutical (Japan)		Debiopharm International SA (Switzerland)	Interventional, measure of safety and tolerability in dose escalation study
Phase I, open-label, multicenter study of AZD4547 in patients with advanced solid tumors (NCT00979134)	979	Oct 2009 / not recruiting, ongoing	AstraZeneca (England)		FGFR inhibitor	Investigate the safety, tolerability and maximum tolerated dose
Study of AZD4547 in patients with FGFR1 or FGFR2 amplified tumors (NCT01795768)	49	Sept 2012 / ongoing - recruiting	Royal Marsden NHS Foundation Trust (England)		FGFR inhibitor	Interventional, assess efficacy within 8 weeks
Phase I, multi-center, open-label, dose escalation study of oral BGJ398, in adult patients with advanced solid malignancies (NCT01004224)	190	Dec 2009 / ongoing - recruiting	Novartis (East Hanover, NJ)		FGFR inhibitor	Interventional, safety, tolerability, pharmacokinetics, pharmacodynamics
Phase I study of JNJ-42756493 in subjects with advanced or refractory solid tumors or lymphoma (NCT01703481)	260	Jun 2012 / ongoing - recruiting	Astex Therapeutic (England)	Janssen Research & Development, LLC (Belgium)	FGFR inhibitor	Interventional, safety, tolerability, pharmacokinetics, pharmacodynamics
Phase I dose escalation study of ARQ 087 in adult subjects with advanced solid tumors (NCT01752920)	120	Dec 2012 / ongoing - recruiting	ArQule (Woburn, MA)		FGFR inhibitor	Interventional, measure of safety and tolerability
Phase I study of LY2874455 in patients with advanced cancer (NCT01212107)	94	Dec 2010 – Feb 2015 / completed	Eli Lilly and Company (Indianopolis, IN)		FGFR inhibitor	Interventional, measure of safety and tolerability
Phase I study of TAS-120 in patients with advanced solid tumors with or without FGF/FGFR-Related abnormalities followed by a Phase II study in patients with advanced solid tumors or multiple myeloma with FGF/FGFR-related abnormalities (NCT02052778)	835	July 2014 / ongoing - recruiting	Taiho Oncology, Inc. (Japan)		FGFR inhibitor	Interventional, measure of safety and tolerability
Phase I, multicenter, open-label study of MFGR1877S in patients with relapsed or refractory multiple myeloma (NCT01122875)	14	Nov 2010 – May 2012 / completed	Genentech, Inc. (South San Francisco, CA)		FGFR3 inhibitor	Interventional, measure of safety and tolerability
Phase I, open-label, dose-escalation study of BAY 1179470 in subjects with advanced, refractory solid tumors (NCT01881217)	63	June 2013 / recruiting - ongoing	Bayer (Whippany, NJ)		FGFR2 inhibitor	Interventional, measure of safety, tolerability, pharmacokinetics, and pharmacodynamics

It has been reported that various molecules can inhibit FGF2 (ligand) activity, binding, or expression in endothelial and tumor cells (Table 5, Figure 4). FGF ligand traps, such as FP-1039, block FGF2 interaction with FGFR (Tables 5 and 6, Figure 4) [85]. A phase I clinical trial was conducted to investigate the safety and tolerability of FP-1039 in advanced solid tumors (Table 6) [86]. FP-1039 is a soluble FGFR1 Fc fusion protein that was engineered to strongly bind all mitogenic FGF ligands. This compound showed promising results and inhibited FGF-mediated cell proliferation and angiogenesis in lung and endometrial cancer models [86]. AGM-1470, miRNA 646, and interferon alpha downregulate FGF2 expression in cancer cells (Table 5, Figure 4). A phase II clinical study was conducted to evaluate the efficacy of pegintron (peginterferon alpha-2b) in patients with stage IV metastatic melanoma overexpressing FGF2 (Table 6) [87]. The results showed that peginterferon alpha-2b suppressed FGF2 levels in 97% of patients with metastatic melanoma to reference range with a median progression free survival (PFS) and overall survival (OS) of 2.0 and 9.7 months, respectively (Table 6) [87].

Other FGF2 antagonists are under investigation for cell line and animal preclinical cancer models. Small molecules, such as sm27, pentosan, and PI-88 as well as proteins such as pentraxin-3 inhibit FGF2 binding to FGFRs (Table 5, Figure 4) [3]. The capability of FGF2 to bind heparin/heparan sulfate indicates that molecules able to interfere with this interaction may act as angiogenesis inhibitors. On this basis, compounds such as suramin, which mimic heparin, can interfere with FGF2 signaling (Table 5). Thalidomide, PAMPS, sirolimus, suramin, and platelet factor 4 inhibit FGF2-induced angiogenesis, while anvirzel inhibits FGF2 export *via* Na^+^/K^+^ pumps (Table 5).

It has been reported that direct inhibition of FGFRs using small molecule inhibitors may be effective in cancer treatment. Several FGFR tyrosine kinase inhibitors (TKIs) are currently in early clinical development (Tables 5 and 6, Figure 4), and many of them exhibit dual specificity for FGFR and VEGFR due to similarity in the ATP binding pocket structure [85]. The first generation of inhibitors were developed as VEGFR and PDGFR inhibitors, but also can inhibit FGFR [88]. These compounds were successful in clinical trials and some of these drugs and drug combinations were subsequently approved by regulatory administrations worldwide for the treatment of different cancers (Tables 5 and 6).

Recently, the U.S. Food and Drug Administration (FDA) granted approval to Lenvima (lenvatinib, developed by Eisai) based on the results of a study on 392 patients with locally recurrent or metastatic, progressive, radioactive iodine-refractory differentiated thyroid cancer (DTC) who were randomly assigned to receive either Lenvima or a placebo. Study results showed that Lenvima-treated participants lived a median of 18.3 months (PFS) compared to a median of 3.6 months for participants who received placebo. Moreover, approximately two thirds of participants treated with Lenvima showed a reduction in tumor size compared to only two percent of participants who received placebo. Similarly, FDA recently approved Iclusig (ponatinib, developed by ARIAD Pharmaceuticals), which is a multi-tyrosine kinase inhibitor for the treatment of adult patients with chronic myeloid leukemia or acute lymphoblastic leukemia (Table 5) [89]. Moreover, the multi-tyrosine kinase inhibitor Votrient (pazopanib, developed by GlaxoSmithKline) was approved by FDA to treat patients with advanced renal cell cancer and patients with advanced soft tissue sarcoma who have received chemotherapy in the past (Tables 5 and 6) [90].

Dovitinib (TKI-258, CHIR-258, developed by Novartis) is a potent VEGFR, PDGFR and FGFR inhibitor. Dovitinib showed promising anticancer and antiangiogenic activity against multiple myeloma and colon cancer models [91, 92]. A phase II trial was conducted to evaluate the safety and tolerability of Dovitinib in relapsed or refractory multiple myeloma patients, who are with or without t(4;14) chromosomal translocation (Table 6). The t(4;14) translocation is observed in approximately 15-20% of multiple myeloma patients, and it is associated with upregulation of FGFR3 and poor prognosis [93, 94]. Upregulation of FGFR3 occurs in nearly 70% of patients with the t(4;14) translocation. Therefore, development of inhibitors such as Dovitinib may show promise in t(4;14) multiple myeloma patients [94]. Further phase II clinical trials were conducted on Dovitinib for treatment of other types of cancers including lung, bladder and gastric cancers (Table 6).

Other investigational multi-kinase inhibitors, such as Brivanib, Orantinib, and Lucitanib, are currently progressing into phases II and III of clinical trials (Tables 5 and 6). Recent clinical trials have assessed the combination of a tyrosine kinase inhibitor with cytotoxic agents (Table 6). Combination strategies that involve the blockade of FGFR signaling with cytotoxic agents have the best clinical outcome for cancer treatment [85]. Recently, Vargatef (nintedanib, developed by Boehringer Ingelheim) was approved in the EU based on the results of a phase III study comparing Vargatef plus docetaxel to placebo plus docetaxel in patients with locally advanced/metastatic non-small-cell lung carcinoma (NSCLC) after first-line therapy in over 1,300 patients in 27 countries [95]. Vargatef is an oral triple angiokinase inhibitor that simultaneously inhibits the VEGFR, PDGFR, and FGFR signaling pathways. The results indicated that treatment with Vargatef and docetaxel significantly extended the median overall survival from 10.3 to 12.6 months for patients with adenocarcinoma compared to placebo and docetaxel, with a quarter of patients surviving for two years or more with minimum side effects [95]. Several clinical trials are ongoing currently with compounds targeted for FGF2; a phase III study was designed to compare the safety and efficacy of a multi-tyrosine kinase inhibitor, masitinib, in combination with bortezomib and dexamethasone to placebo in combination with bortezomib and dexamethasone in patients with relapsing multiple myeloma. Masitinib is an orally active and bioavailable compound that is a weak inhibitor of FGFR3 [96].

More recently, second-generation inhibitors targeting FGFRs with high selectivity have been developed (Table 5, Figure 4). For example, Debio 1347, developed by Debiopharm Group, a Swiss-based global biopharmaceutical company, is an orally bioavailable inhibitor of FGFRs 1-3 that inhibits FGFR-mediated signal transduction pathways and consequently tumor cell proliferation and angiogenesis [97]. Debio 1347 will be used for personalized cancer treatment through the development of a companion diagnostic. It is currently being evaluated in Europe and the USA in a phase I trial to evaluate its safety and tolerability in patients with advanced solid tumors displaying alterations in FGFRs 1, 2, or 3 genes (Tables 5 and 6) [97]. Similarly, AZD4547, developed by AstraZeneca, is an orally tolerated inhibitor of FGFRs 1-3 [98]. AZD4547 inhibits FGFR kinase activity and tumor growth *in vitro* and *in vivo* [98]. This compound is currently being tested for safety and efficacy in different clinical trials against advanced tumors (Table 6). Other small molecule FGFR inhibitors, such as BGJ398, JNJ-42756493, ARQ 087, LY2874455, and TAS-120, are currently under clinical investigation for advanced, relapsed or refractory tumors and most of these trials are still recruiting patients (Tables 5 and 6). Other selective small molecule tyrosine kinase inhibitors, such as PD 161570, PD 173074, PD 166285 dihydrochloride, PD 166866, FIIN hydrochloride, SU 5402, and SSR128129E are currently being tested for their antitumor activity on cell lines and *in vivo* preclinical models (Table 5). Monoclonal antibodies specifically targeting particular FGFR isoforms are also being developed. MFGR1877S (developed by Genentech) is a human anti-FGFR3 monoclonal antibody that inhibits tumor progression of bladder carcinoma and multiple myeloma xenografts in mice by antagonizing FGFR3 signaling [99]. A phase I clinical trial was conducted to evaluate the response to MFGR1877S in patients with relapsed or refractory multiple myeloma (Tables 5 and 6). Similarly, BAY 1179470 (developed by Bayer) is a human anti-FGFR2 monoclonal antibody. BAY 1179470 showed antitumor activity in gastric cancer xenograft models with high FGFR2 expression [100]. The anti-FGFR2 antibody BAY 1179470 is currently in Phase I testing in subjects with advanced, refractory solid tumors (Tables 5 and 6)

## CONCLUSIONS

FGF2 is frequently dysregulated in cancer, especially in advanced stages of disease. The upregulation of FGF2 or FGFRs can promote resistance to chemotherapy. FGF2 is currently being evaluated in clinical studies as a potential predictive biomarker for hematological and solid tumors. In addition, FGF2/FGFR inhibitors are being developed and evaluated as monotherapy or as part of a combination therapy for the treatment of different types of cancer. Identifying patients with advanced, relapsed or refractory cancers that would benefit from FGF2/FGFR signaling inhibition will allow for better treatment options of those patients in the era of personalized medicine.
